# Investigating Falls and Rehabilitation Use in Older Wheelchair Users from the National Health and Aging Trends Study

**DOI:** 10.1093/geroni/igaa057.3335

**Published:** 2020-12-16

**Authors:** Qiong Nie, Laura Rice, Jacob Sosnoff, Wendy Rogers

**Affiliations:** University of Illinois at Urbana-Champaign, Champaign, Illinois, United States

## Abstract

Falling is a serious health concern among older wheelchair users as falling may result in functional impairment, morbidity, and mortality. Researchers have examined prevention strategies of falls. However, little is known about the prevalence of falls and rehabilitation service use among wheelchair users over age 65. We analyzed a population-based data set – the National Health and Aging Trends Study– to describe trends over time of wheelchair use among older adults who completed a sample interview in this study; we further identified 417 community-dwelling older wheelchair users in wave 5, described prevalence of falls and rehabilitation service use among them and investigated indicators of fall-related rehabilitation service utilization using multinomial logistic regression. The results indicated that there was an increasing trend of wheelchair use from 2011 (7.2%) to 2019 (10.6%). Falling was a common problem (56.1%); however, only 12.1% received rehabilitation service addressing the problem of falls. Around half of rehabilitation service users reported that their functioning improved, but the effect was not lasting. Age (74-79 years old: odds ratio [OR] =2.72, 95% confidence interval [CI], 1.01-7.33; 80-84 years old: OR= 2.93, 95% CI, 1.19-7.25) and balance or coordination problem (OR= 4.70, 95% CI, 1.84-12.06) were associated with receiving fall-related rehabilitation. These data suggest that we need to understand more about the underuse of the rehabilitation services, ensure that older wheelchair users are equally utilizing the services to prevent falling and improve the long-lasting effect of the services.

